# Apple quality identification and classification by image processing based on convolutional neural networks

**DOI:** 10.1038/s41598-021-96103-2

**Published:** 2021-08-17

**Authors:** Yanfei Li, Xianying Feng, Yandong Liu, Xingchang Han

**Affiliations:** 1 School of Mechanical Engineering, Shandong University, Jinan, 250061 Shandong China; 2 Key Laboratory of High Efficiency and Clean Mechanical Manufacture of Ministry of Education, Shandong University, Jinan, 250061 Shandong China; 3 Shandong Academy of Agricultural Machinery Sciences, Jinan, 250100 Shandong China

**Keywords:** Electrical and electronic engineering, Mechanical engineering

## Abstract

This work researched apple quality identification and classification from real images containing complicated disturbance information (background was similar to the surface of the apples). This paper proposed a novel model based on convolutional neural networks (CNN) which aimed at accurate and fast grading of apple quality. Specific, complex, and useful image characteristics for detection and classification were captured by the proposed model. Compared with existing methods, the proposed model could better learn high-order features of two adjacent layers that were not in the same channel but were very related. The proposed model was trained and validated, with best training and validation accuracy of 99% and 98.98% at 2590th and 3000th step, respectively. The overall accuracy of the proposed model tested using an independent 300 apple dataset was 95.33%. The results showed that the training accuracy, overall test accuracy and training time of the proposed model were better than Google Inception v3 model and traditional imaging process method based on histogram of oriented gradient (HOG), gray level co-occurrence matrix (GLCM) features merging and support vector machine (SVM) classifier. The proposed model has great potential in Apple’s quality detection and classification.

## Introduction

Apples are very popular agricultural products with high nutritional value^1^. After years of development, China has become the world’s largest apple producer, with apple planting area and yield accounting for more than 50% of the world. One of the important reasons affecting the export of apples is that the quality of the apples is rather spotty. With increased attention for fruits of high quality and safety standards, the demand for automatic, accurate and fast quality identification continues to grow^2^. The exponential population spurt threatens to reduce levels of food security as time progresses^3,4^. Therefore, defective apples should be precisely detected and automatically weeded out before they are sold in the market.

Bio-molecular sensing technology, hyperspectral imaging techniques, multispectral imaging, and traditional machine vision technology are effective detection methods for detecting quality fruits. Low et al.^5^ constructed an electrochemical impedance genosensing platform based on graphene/zinc oxide nanocomposite to enhance sensitivity of plant disease detection. Wijesinghe et al.^6^ proposed a detection method based on bio-photonic technology to improve sensitivity of apple diseases. Hyperspectral imaging techniques combine the advantages of traditional spectroscopy technology and imaging technology^7^. Due to the rich data information, hyperspectral imaging technology has developed into the effective method for quality identification of fruit. Zhang et al.^8^ presented the detection algorithm based on successive projections algorithm, particle least square discriminant analysis and minimum noise fraction (SPA-PLS-DA-MNF) by using hyperspectral imaging techniques and spectral analysis for the rottenness apple detection with the accuracy of 98%. The automated detection system based on near-infrared (NIR) coded spot-array structured light was proposed recently for detection of defective apples with overall identification accuracy of 90.2% for three categories^9^. Keresztes et al.^10^ proposed hyperspectral imaging (HSI) in the shortwave infrared (SWIR) system to detect bruise region for ‘Jonagold’, ‘Kanzi’ and ‘Joly Red’ apples. Partial least squares-discriminant analysis (PLS-DA) model was also employed to discriminate between sound, bruised, glossy and stem regions and reached to 94.4% accuracy. Zhang et al.^11^ introduced full-wavelength model to detect blueberry bruising using hyperspectral transmittance imaging with accuracy of 81.2%. With the research of hyperspectral technology, multispectral technology is favored by researchers in the field of fruit quality identification. Unay et al.^12^ presented automatic classification system to grade bi-colored apples into two-category and obtained 93.5% overall accuracy by multispectral imaging techniques. Li et al.^13^ proposed multispectral algorithm to detect early decay in citrus. Zhang et al.^14^ proposed image recognition method to inspect damage, insect damage, bruises, decay apples by multispectral imaging with overall detection accuracy of 91.4%. Pontes et al.^15^ used Mass spectrometry imaging for orange trees disease detection. A number of imaging models have been effectively used in fruit quality identification, such as reflectance^16^, transmittance^17^, fluorescence^18^, and Raman^19^, but the improvement of recognition accuracy is still a challenge. Although hyperspectral and imaging technology has broadened the application range of machine vision, the huge amount of data in hyperspectral images affected the efficiency of detection. The uneven brightness exists in the hyperspectral image, which still interfered with the detection of apple surface defects.

Most quality inspections of fruits can be achieved with traditional machine vision based on color cameras. Li et al.^20^ realized the automatic detection of citrus fruit surface defects based on brightness transformation and image ratio algorithm, and achieved 98.9% detection rate. Dubey et al.^21^ presented a method using color, texture and shape features from images and reached to 95.94% disease detection accuracy. Moallem et al.^22^ introduced a computer vision-based algorithm to identify the defect in apple and obtained accuracy of 92.5% and 89.2% for healthy and defected apples using SVM. Bhargava and Bansal^23^ proposed fruit grading system with SVM to grade mono-colored apples into healthy or defected quality categories by textural, geometrical, and statistical features. This system was tested by two datasets with maximum accuracy of 96.81% and 93.00%, respectively. Bhargava and Bansal^24^ presented a system to detect the quality of apple, avocado, banana, and orange by fuzzy C-means clustering with accuracy of 95.72% using SVM. But the above methods are only for defect detection, and do not carry out more detailed classification. With color, texture and shape features are often used to identify disease of fruit, the identification rate of those works is highly dependent on feature extraction.

Recently, artificial intelligence methods represented by deep learning, especially convolutional neural networks (CNN), have achieved a series of important results in the field of fruit quality detection and classification. To overcome issues of above methods, some researchers used deep learning methods for quality identification in agriculture. Sun et al.^25^ employed pixel-based convolutional neural network to identify early decay peaches and achieved 97.6% detection rate. Barman et al.^26^ constructed Self-Structured classifiers by CNN to grade citrus leaf into three categories with training accuracy and validation accuracy of 98% and 99%, respectively. Fan et al.^27^ presented a deep learning architecture based on CNN to detect defective apples and obtained training accuracy and testing accuracy of 96.5% and 92%, respectively. However, above references not used mainstream frameworks to verify and compare on his own dataset, and training time of model was not considered. Thus, it is particularly important to find a robust, accurate and fast model for apple quality identification and classification.

The overall goal of present study was to evaluate potential of proposed model based on CNN for the accurate and fast grading of apple quality. The specific objectives of the work were to: (1) develop and train proposed CNN-based identification architecture for apple quality classification using apple samples; (2) compare the overall performance of proposed CNN-based architecture, Google Inception v3 model (mainstream framework), and HOG/GLCM + SVM (traditional method); and (3) evaluate the performance of three models for grading apples into three quality categories using independent dataset.

## Materials and methods

This study is in compliance with relevant institutional, national, and international guidelines and legislation.

### Build dataset

**Figure 1:**
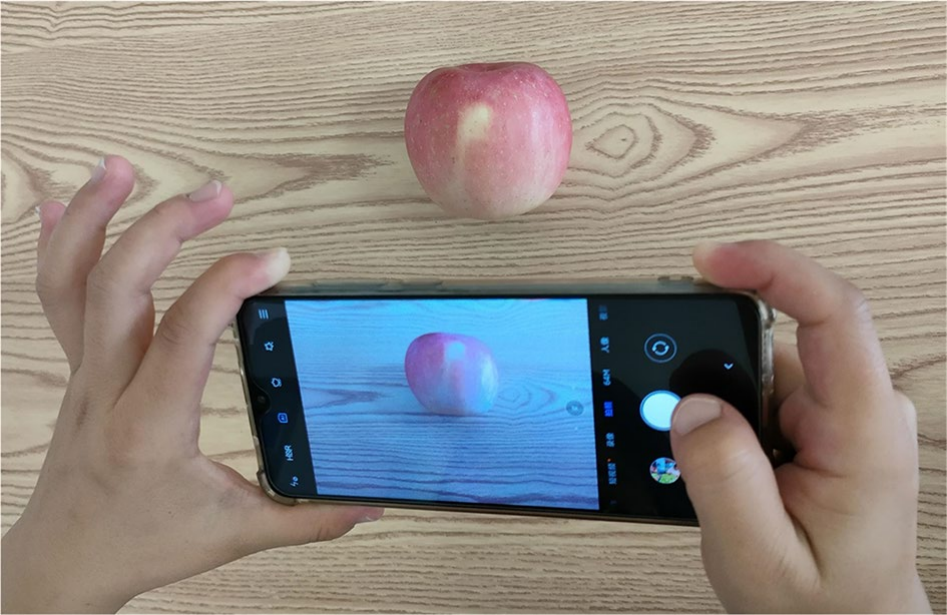
The process of images capturing using mobile camera.

It is important that richer information features of the apple data were constructed for the model to identify targets. ’Yantai Red Fuji’ apples were purchased form RT-MART supermarket in Jinan, Shandong Province. In order to increase the accuracy and robustness of recognition model, the time, angle and light intensity of the image collection were different. In total, 3600 original images (a resolution of 3120 × 4160) were obtained by the same mobile camera (13 mega-pixels, F-Stop = f/2, Exposure time = 1/50 s, ISO speed = 151, Focal length = 4 mm, Redmi Note 4X, China)^28^, which were then divided into three categories of premium, middle and poor grade. The process of images capturing using mobile camera was shown in Fig. 1.

**Figure 2:**
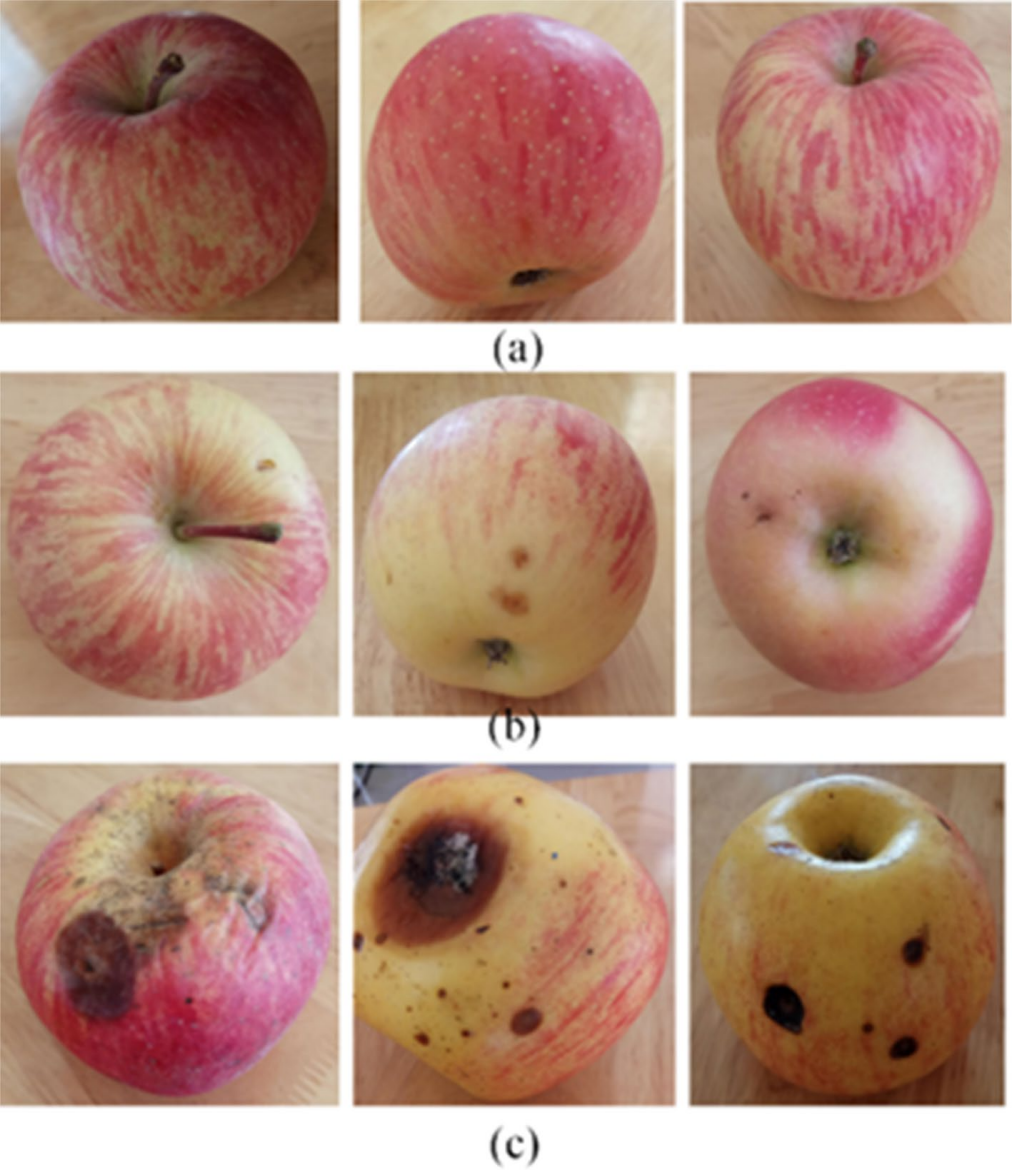
A few of images of premium (**a**), middle (**b**) and poor (**c**) grade apples.

The problem of insufficient size of the training dataset has been solved by techniques of data augmentation^29^. In the case of insufficient dataset, a direct and effective way of data enhancement technology can increase the diversity of training samples, improve the robustness of the model, and avoid overfitting. Changing the training samples can reduce the dependence of the model on some attributes or features using data augmentation technology. The more valuable data based on existing dataset were created by data augmentation strategy. As a result, the performance and robustness of model were improved. In order to reduce the calculation time of data augmentation, the pixels of the original pictures were zoomed out images with a quarter of the original images (a resolution of 780 × 1040). A few sample images in each class were shown in Fig. 2.

**Figure 3:**
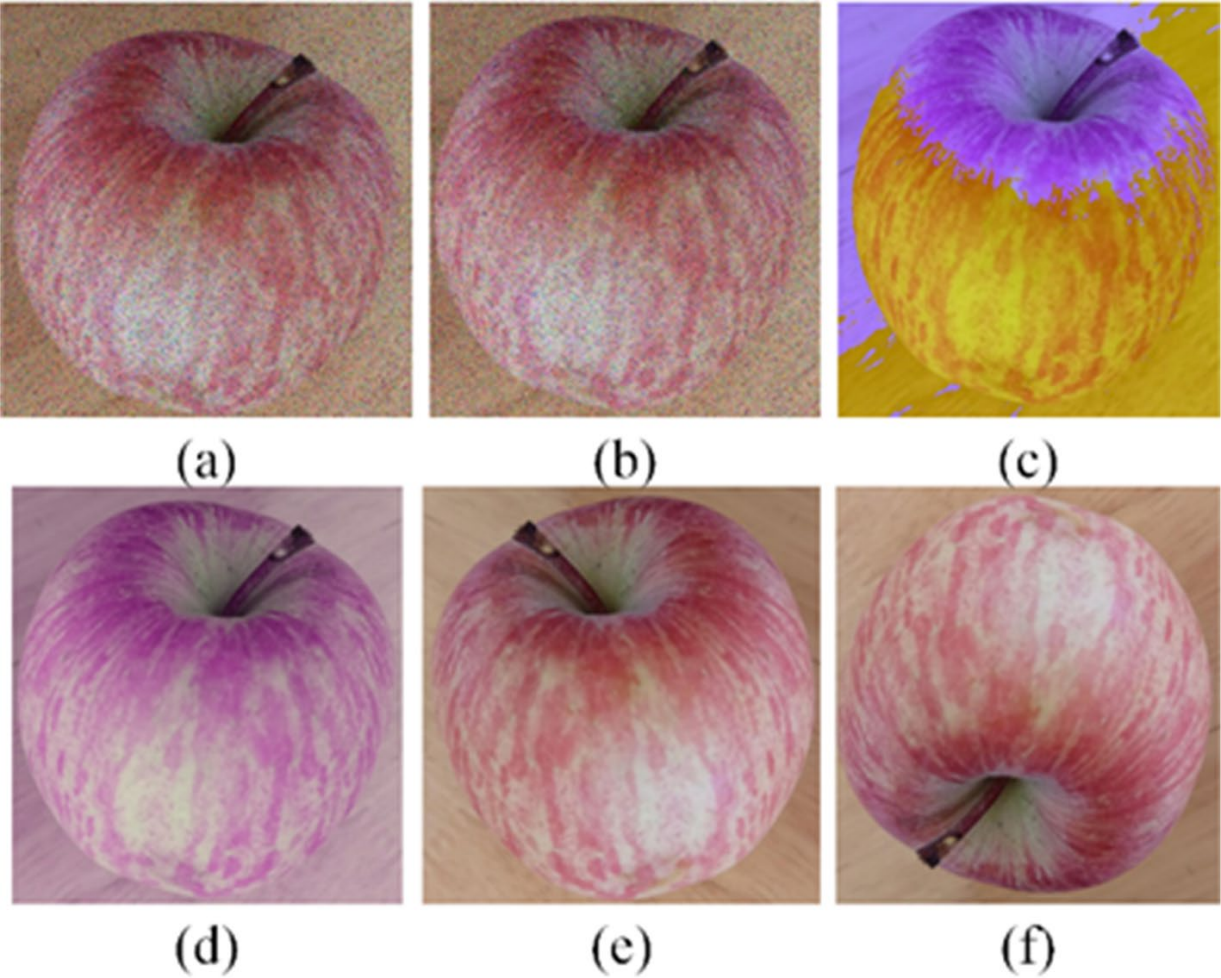
Representative augmented images: (**a**) increasing salt and pepper noise; (**b**) increasing Gaussian noise; (**c**) brightness; (**d**) darkness; (**e**) flips; (**f**) rotation at 180°.

In this work, several augmentation techniques without changing the semantics of the images were applied in each scaled-down image such as increasing salt and pepper noise, increasing Gaussian noise, flips, rotation, brightness, and darkness operation. The noise density of salt and pepper noise was 0.3. The operation of increasing Gaussian noise prevented effectively the neural network from fitting all the features of the input image. The manipulation of horizontal flips was used. The percentage of brightness and darkness was respective 1.5 and 0.9. The manipulation of rotation was used to each sub-image, which could generate other five sub-images at 60°, 120°, 180°, 240°, and 300°. Finally, 36,000 apples sub-images were obtained. Augmented sub-images were shown in Fig. 3. The training and validation sets were independent and randomly sampled form 36,000 apple sub-images dataset with proportion of 80% and 20% (28,800 for training, 7200 for validation).

### Overall identification architecture

A typical convolutional neural network (CNN) consists of input layer, convolutional layer, full connection layer and output layer. The advantage of convolution is local receptive fields and shared weights, rather than the way of all neurons are connected in the artificial neural network (ANN). In this way, the training parameters of the network were substantial decreased^30^. A CNN-based identification architecture, which was composed of an input layer, 6 convolutional layers (convolution and pooling operations), 2 full connection layers and an output layer, was developed for apple quality recognition. The specific configurations of the proposed model were shown in Table 1.

**Table 1:** Detailed configurations and properties of the proposed model.

Layer	Operation	Kernel shape	Number of Kernel	Stride	Number of neurons	Number of training parameters	Number of connections
Conv1	Convolution	5 × 5	8	1	–	208	8,998,912
	Pooling	3 × 3	–	2	–	24	865,280
Conv2	Convolution	3 × 3	16	1	–	1168	12,633,088
	Pooling	3 × 3	–	2	–	48	432,640
Conv3	Convolution	1 × 1	32	1	–	1168	1,470,976
Conv4	Convolution	3 × 3	64	1	–	18,496	50,013,184
	Pooling	3 × 3	–	2	–	192	432,640
Conv5	Convolution	3 × 3	64	1	–	46,460	31,204,160
	Pooling	3 × 3	–	2	–	240	115,200
Conv6	Convolution	3 × 3	64	1	–	466,144	6,644,736
	Pooling	3 × 3	–	2	–	192	23,040
Fc1	Full connection	–	–	–	256	590,080	590,080
Fc2	Full connection	–	–	–	256	65,792	65,792
Output	Softmax	–	–	–	3	–	–

In the first convolutional layer, in order to acquire high-level features, convolutional kernel shape was 5 × 5, the number of convolution kernels were 8, the stride of the convolution kernel was 1. After the convolution operation, the size of the input image did not change, but the dimension was increased from 3 to 8 because of the convolution mode of SAME PADDING was selected. During the convolution operation, the original features of the input images were not lost using SAME PADDING. In order to reduce the dimensions of images, pooling layer kernel shape was 3 × 3, the stride of the pooling kernel was 2. Inspired by the Hebbian theory^31^, convolutional kernel shape of convolution layer 3 was 1 × 1. The convolution kernel (1 × 1) could connect highly correlated features in the same spatial location but different channels. This leaded to a large difference in feature information between adjacent pixels. Therefore, pooling was not used in this layer.

### Network updating process

**Figure 4:**
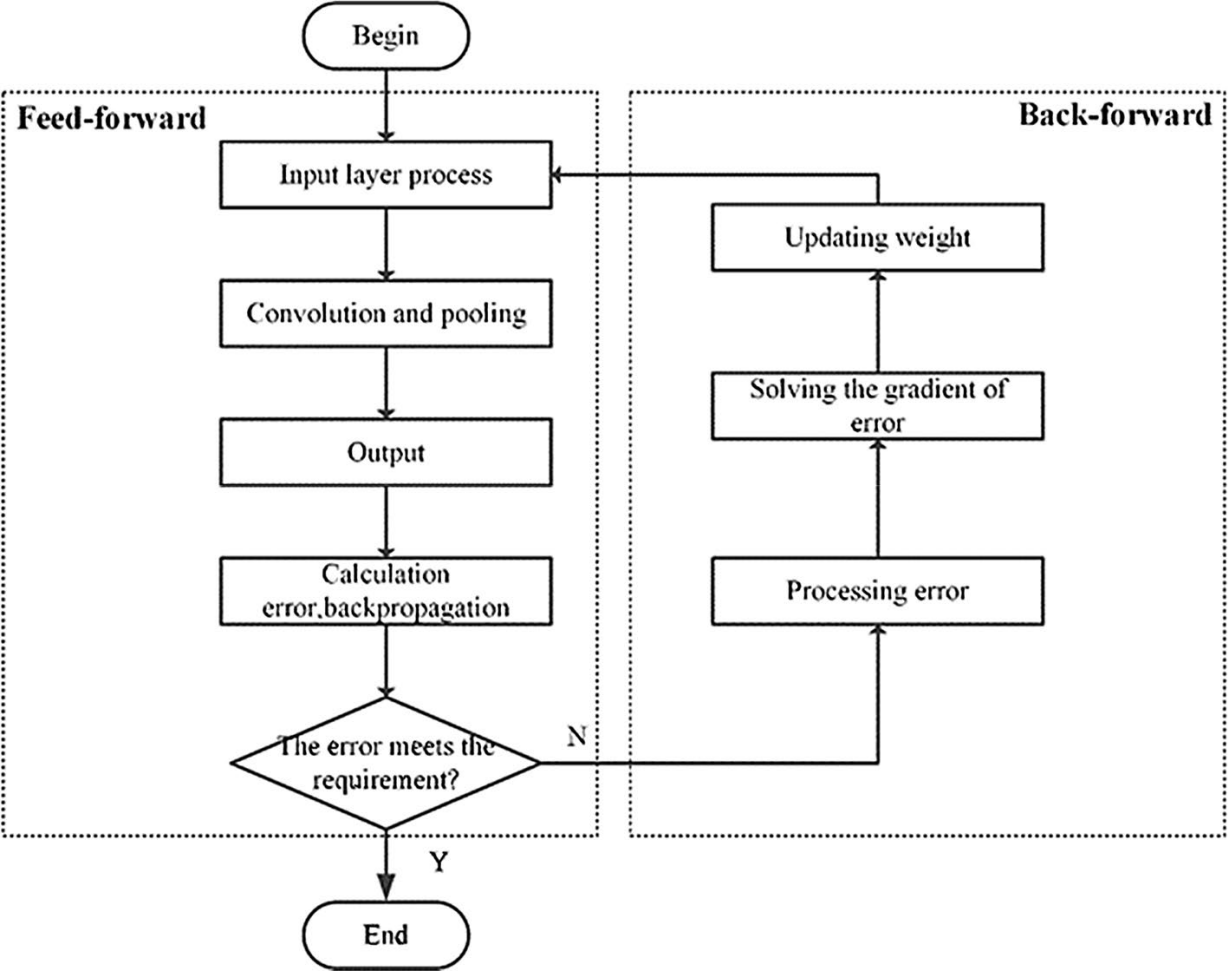
Flowchart of the algorithm for proposed apple identification model.

The proposed model was trained using the error back propagation algorithm, which was divided into two processes. The flowchart of the updating process for proposed model was shown in Fig. 4.

#### Feed-forward propagation

When it comes to convolution operations, it was inseparable from the concept of discrete convolution in mathematics. Discrete convolution was defined as Eq. (1).

1 $$ y(n) = \sum\limits_{i = - \infty }^{\infty } {x(i) \cdot h(n - i)} , $$

where *x*(*n*) and *h*(*n*) represent discrete sequences respectively. *y*(*n*) represents a new sequence obtained by convolution.

The convolution of the two-dimensional discrete function *f* (*x*, *y*) and *g* (*x*, *y*) was defined by Eq. (2).

2 $$ f(x,y) \otimes g(x,y) = \sum\limits_{i}^{\infty } {\sum\limits_{j}^{\infty } {f(i,j) \cdot g(x - i,y - j)} } . $$

The output of convolution layer neuron were defined as Eq. (3).

3 $$ f(x) = act\left( {\sum\limits_{i,j}^{n} {x_{ij} \theta_{(n - i)(n - j)} + b} } \right), $$

where *act* represents activation function.$x_{ij}$,$\theta$ and *b* represents *i* row and *j* column of pixels, kernel shape and offset value, respectively.

The output of convolution layer and pooling layer were obtained by Eqs. (4) and (5), respectively.

4 $$ O_{j} = \phi \left( {\sum\limits_{Z} {X_{Z} \theta_{j} + b_{j} } } \right), $$

5 $$ S_{j} = f(P(O_{j} ) + b_{j} ), $$

where $S_{j}$ represents pooling output of the* j* feature. *f*, *P* and *b* represent activation function, down-sampling function and offset value, respectively.

After the processing of the pooling layer, a series of feature maps were obtained. Take out the pixels in order from the feature maps and arranged them into a vector. This method was called rasterization. The definition of rasterization was shown as Eq. (6).

6 $$ O_{k} = [x_{111} ,x_{112} , \ldots ,x_{11n} ,x_{121} ,x_{122} , \ldots ,x_{12n} , \ldots ,x_{1mn} ,x_{2mn} , \ldots ,x_{jmn} ], $$

where $O_{k}$ represents rasterized vector. Finally, the rasterized vectors were input to the fully connected layer and the classification results were obtained.

#### Back-ward propagation

Backward propagation was mainly the propagation of errors. The error vector $\delta_{k}$ of rasterization was defined as Eq. (7).

7 $$ \delta_{k} = [\delta_{111} ,\delta_{112} , \ldots ,\delta_{11n} ,\delta_{121} ,\delta_{122} , \ldots ,\delta_{12n} ,\delta_{1mn} ,\delta_{2mn} , \ldots ,\delta_{jmn} ]. $$

The vector error of the pooling layer and convolution layer were shown as Eqs. (8) and (9), respectively.

8 $$ \Delta_{k} = \{ \Delta_{1} ,\Delta_{2} , \ldots ,\Delta_{m} \} , $$

9 $$ \Delta_{p} = F(\Delta_{k} ), $$

where *m* and *p* represent the number of pooling and convolution, respectively. $\Delta_{k}$ and $\Delta_{p}$ represent vector error of the pooling layer and convolution layer, respectively. *F* represents Up-sampling Function.

The weight update of a certain region C in the convolutional layer *q* was calculated by Eq. (10).

10 $$ \frac{\partial E}{{\partial \theta_{q} }} = rot180\left( {\left( {\sum\limits_{p} {O_{p} } } \right)_{j} rot180(\Delta_{q} )} \right), $$

where *E*,$\theta_{q}$ represent error function, weight, respectively. $rot180$ represents the matrix that was rotated 180°.$O_{p}$, $\Delta_{q}$ represent Pooling output, sum of all bias gradients, respectively. The final propagation error $\Delta_{p}$ was defined as Eq. (11):

11 $$ \Delta_{p} = \left( {\sum\limits_{q \in C} {\Delta_{q} rot180(\theta_{q} )} } \right) \cdot O_{p} . $$

### Apple quality identification using other two methods

**Figure 5:**
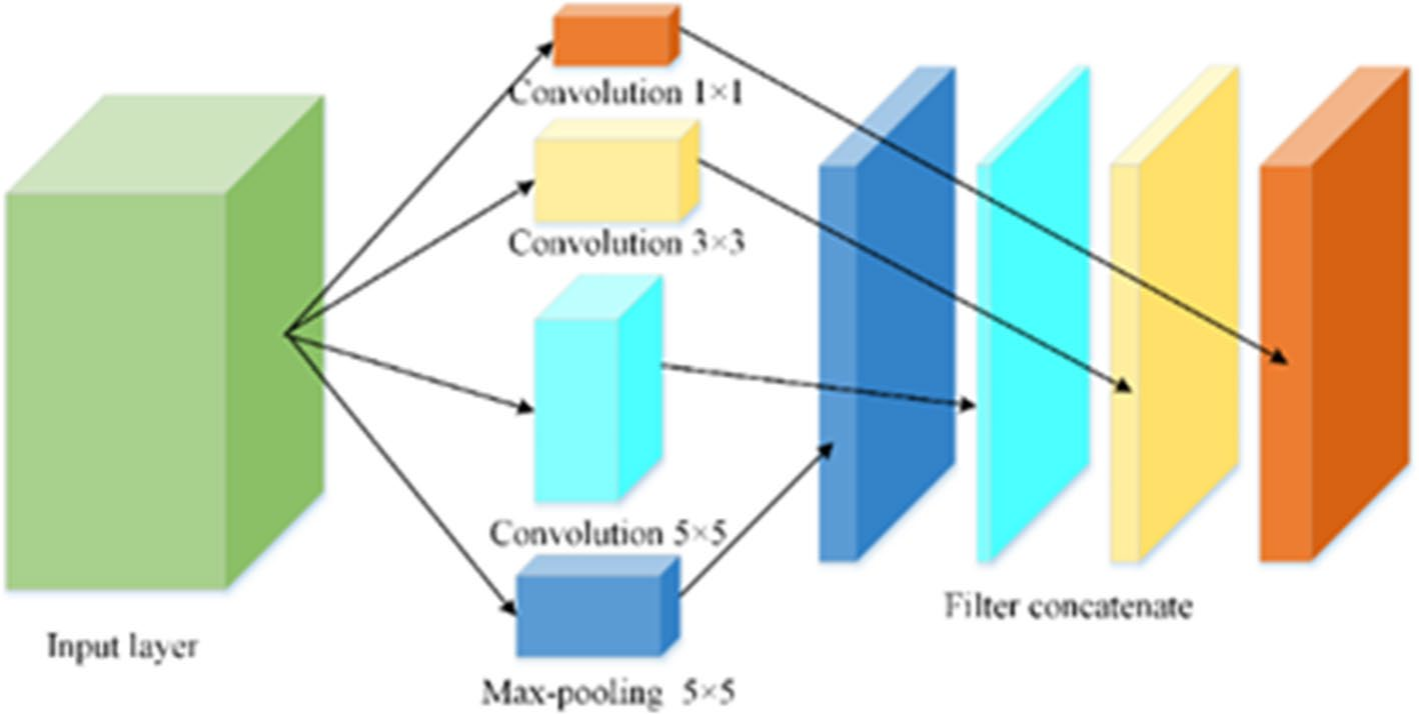
Diagram of internal structure of inception module.

In order to compare the performance of apple quality identification by proposed CNN-based architecture, the Google Inception-v3 model^32^ was used for quality identification under the same dataset. In this model, the fully connected layer was replaced by global average pooling for reducing the computational complexity. The main contribution of Google Inception-v3 was the Inception module. Inception v3 was the most classic and stable model of Google Net, it contained 10 inception modules. The accuracy of the model was improved by increasing the depth and width of the network and reducing parameters in Inception module. The structure of Inception was composed of convolution operations corresponding to 1 × 1, 3 × 3, and 5 × 5 convolution kernels and pooling operations corresponding to 3 × 3 filters, which increased the adaptability of the network to scale. The structure diagram of Inception was shown in Fig. 5.

**Figure 6:**
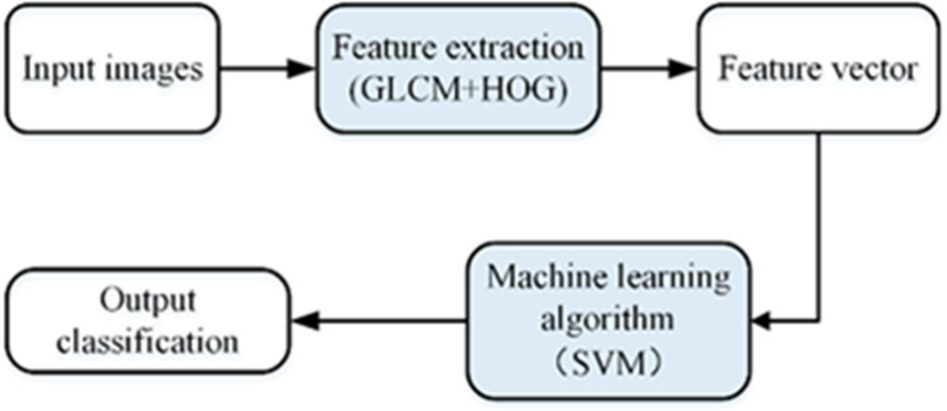
Flowchart of traditional method.

Similarly, a traditional method was applied for apple quality identification in this study. The traditional method was the work of converting images data from two-dimensional gray space to target pattern space. The result of classification was that the image was divided into several subareas of different categories according to different attributes. Generally, the difference in properties between different image regions after classification should be as large as possible, and the internal properties of the regions should be stable. The flowchart of traditional method was shown in Fig. 6.

The comprehensive information of the image gray level related to direction, adjacent interval, and amplitude of change were reflected by the GLCM of apple image, which were the basis for analyzing the local patterns of the image and arrangement rules. The texture description method of GLCM studied the spatial dependence of gray levels in image texture^33^.

The apple images features were extracted by calculating and counting the gradient direction histogram of the local area of the images using HOG method. In order to improve the robustness of HOG features to change in illumination, square root Gamma compression was used to achieve the normalization. The normalized images were convolved using one-dimensional discrete differentiation [− 1,0,1] to obtain the gradient component in the horizontal and vertical direction. According to the horizontal vertical gradient of the current image pixel, the gradient amplitude and gradient direction of the pixel were obtained, and the gradient direction histogram was also constructed.

## Results and discussions

### Experimental details and results of proposed CNN-based architecture

The methods of parameters selection were used in a variety of ways in the literatures of training CNN model. However, some basic principles still need to be observed in the parameter setting. In this work, Cross entropy function was selected as loss function. Adam Optimizer was selected as optimizer since Adam algorithm made the update of weights and offsets more stable. The size of the input images was set to 208 × 208 × 3. The maximum number of training step was set to 3001 taking into account the total number of data sets and the number of layers of the architecture. In the training process, the learning rate is too large, which makes the network unable to converge, and the learning rate is too small to make the function converge slowly^34^. Therefore, learning rate was set to 0.0001 in this work. The training batch size was selected as 20.

**Figure 7:**
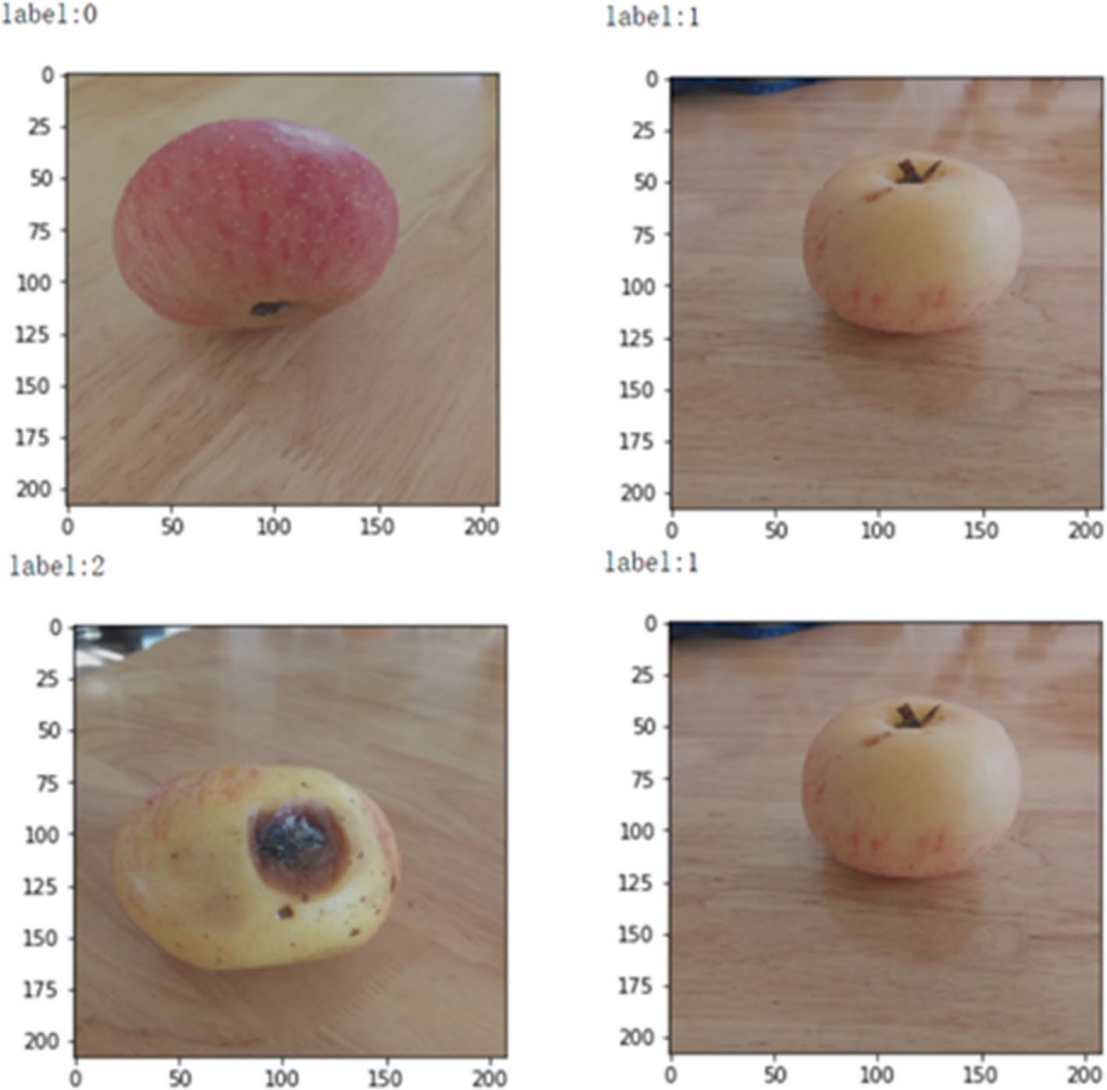
Representative preprocessed sub-images for proposed model.

The sub-images from the dataset need to be processed and recognized by the learning model before model training. Figure 7 showed that two batches of images were randomly generated by preprocessing (label 0, label 1, label 2, label 1). Label 0, label 1, and label 2 represent premium grade, middle grade, and poor grade, respectively.

**Figure 8:**
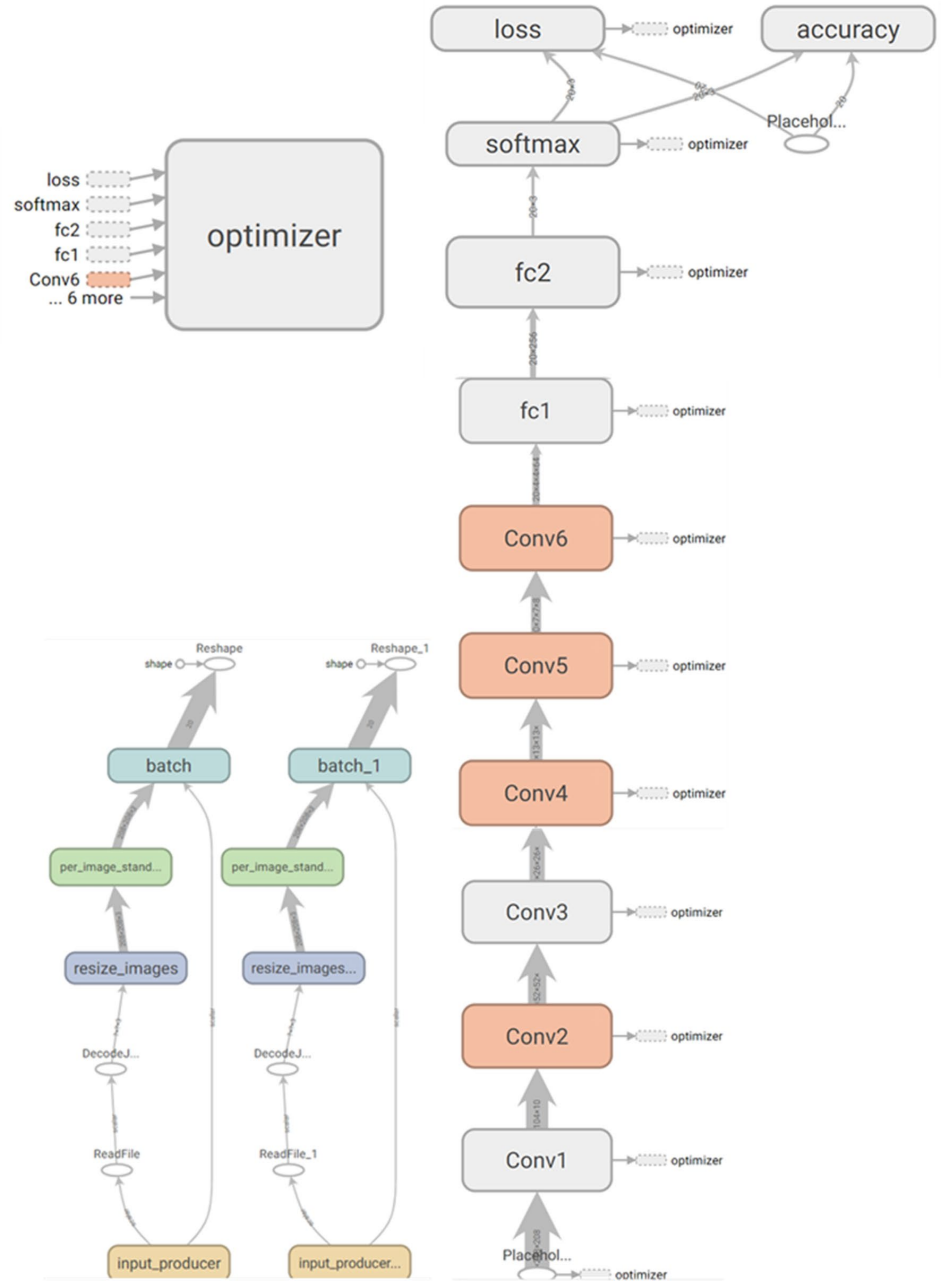
Visual architecture of the proposed model.

After preprocessing, the proposed CNN-based architecture was implemented by TensorFlow (1.8.0 CPU only) on Windows system with Intel i7-10700@2.9 GHz and 16 GB RAM using Python Language and was visualized by TensorBoard (1.8.0). The proposed model was fully shown in Fig. 8.

**Figure 9:**
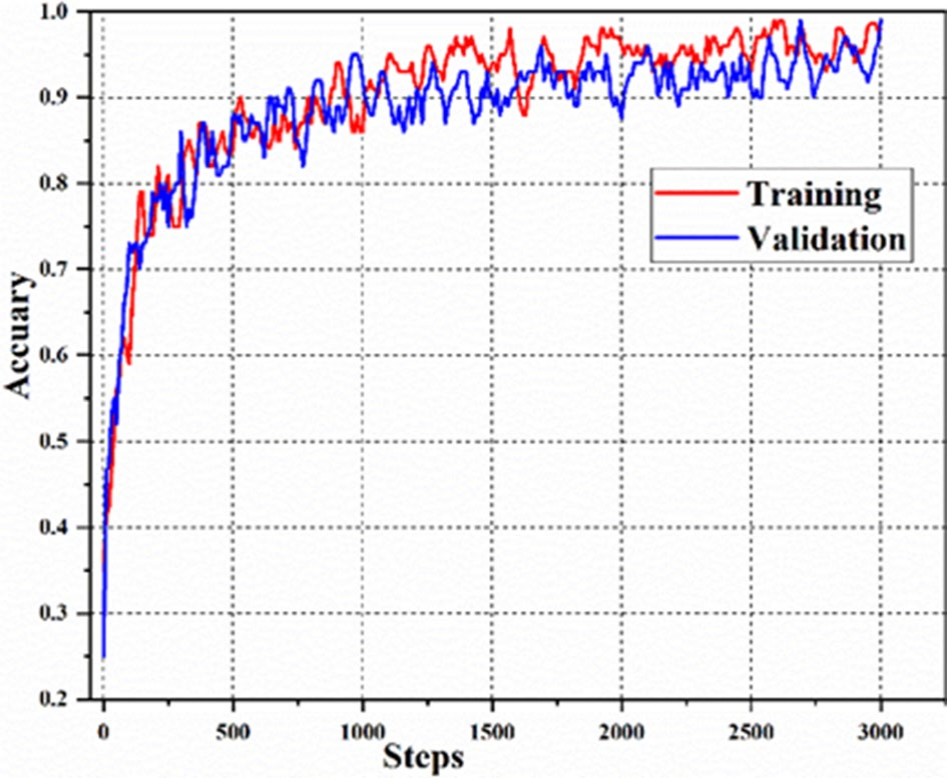
Training and validation accuracy curves of the proposed model.

The training and validation accuracy curves of the proposed model were shown in Fig. 9. The whole training process achieved satisfactory results by optimizing weights and bias values at each step. The training time of proposed model was 27 min. The accuracy curves of training and validation sets increased exponentially at 1000 steps and held steady around 96% and 93% after about 2000 steps, respectively. It showed that there is no or slight overfitting in proposed model. After 3001 steps, the trained proposed model and it all parameters were saved. It also indicated that the recognition accuracies in training and validation sets reached their maximum at the 2590th and 3000th step (99% and 98.98%), respectively. The corresponding losses were 0.554 and 0.589 for training and validation, respectively. The training results demonstrated that the proposed architecture has a great potential for apple quality identification.

### Performance of Google Inception v3 model for apple quality identification

**Figure 10:**
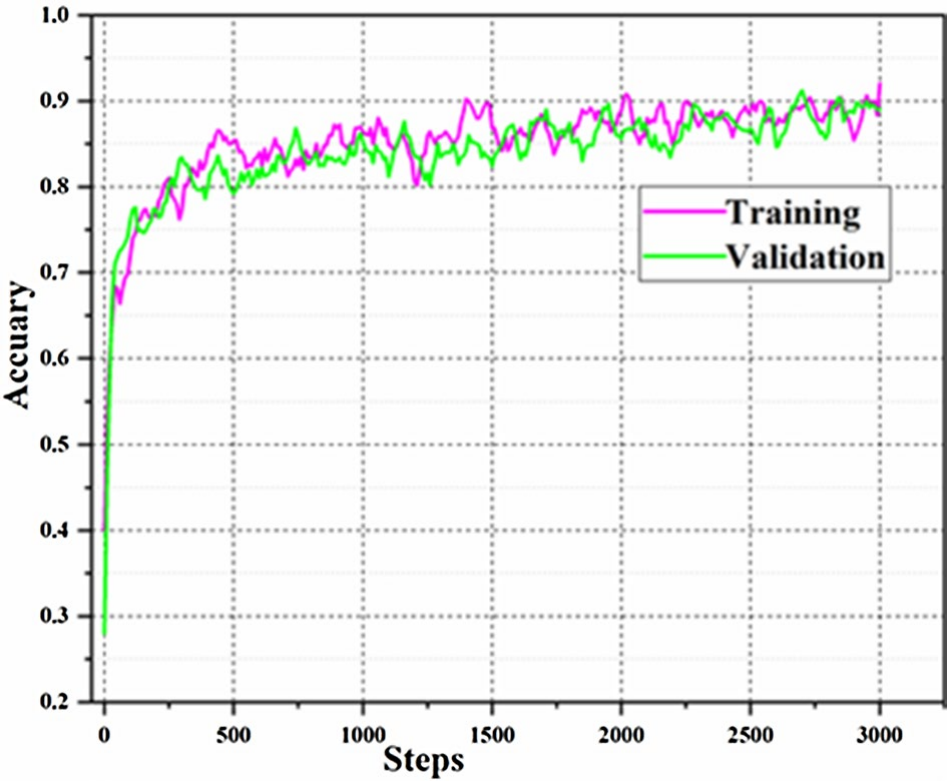
Training and validation accuracy curves of the Google Inception v3 model.

Although Google Inception v3 model has achieved very good recognition results on the ImageNet Large Scale Visual Recognition Challenge (ILSVRC), the process of training all the parameters of the model were relatively time-consuming with the huge training datasets applied. A trained Google Inceptionv3 model was downloaded from Github. In order to save time and prevent overfitting, the parameters of the convolutional layers and the pooling layers were not changed and only the last layer of the model was trained during the training process. Parameters setting for Google Inception v3 model were similar to proposed model. The greatest accuracy generated by Google Inceptionv3 model in training and validation were 92% and 91.2% at the 3000th and 2700th step, respectively, as shown in Fig. 10. In addition, the training time for Google Inception v3 model was 51 min. Although the accuracy curves of the Google Inception v3 model fluctuated less than the proposed model, the proposed model can achieve much better accuracy in apple quality identification than the Google Inception v3 model.

### Performance of traditional methods for apple quality identification

**Figure 11:**
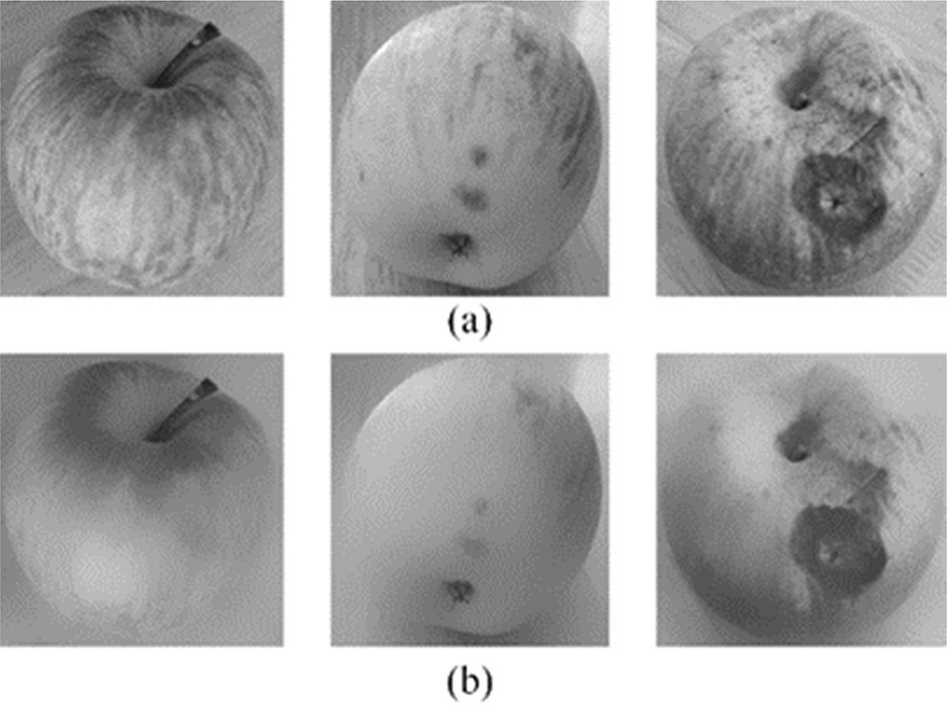
Representative preprocessed sub-images for traditional method: (**a**) grayscale; (**b**) sub-images processed by bilateral filtering.

In order to achieve the effect of image enhancement, the gray range of the sub-images were changed by weighted mean method. After the grayscale processing, bilateral filtering was applied in this work which reduced sharp changes and noise of image grayscale. Bilateral filtering was a non-linear filter^35^ that retained the relationship between pixels in the spatial distance at the time of sampling and that increased the correlation between pixels to maintain edge features. In the preprocessing of the apple sub-images, the neighborhood diameter was set to 90, sigmaColor and sigmaSpace were set to 75. Bilateral filtering could preserve the detailed contour information of the apple. The results of preprocessing were shown in Fig. 11.

After preprocessing, the local and structural features of the apple images were extracted using GLCM and HOG methods, respectively. In order to improve recognition efficiency, four texture parameters of Angular Second Moment (Asm), Entropy (Ent), Contrast (Con) and Correlation (Cor) were adopted as texture features. Asm was used to calculate the uniformity of the images. Ent described the amount of information of the apple images. Con reflected the clarity of the images and the depth of the textures. Cor measured the similarity of the gray levels of the images in the row or column direction. Their average and variance were used as the local extracted features.

**Figure 12:**
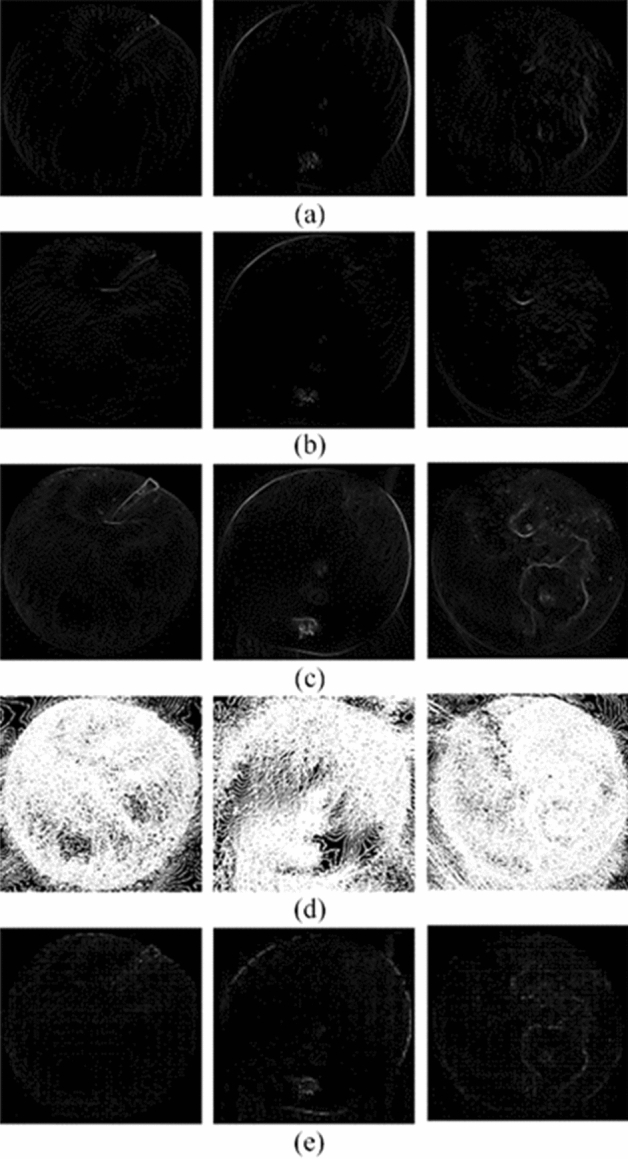
The results of structural feature extraction using HOG for apple images: (**a**) x-direction gradient; (**b**) y-direction gradient; (**c**) gradient magnitude; (**d**) gradient direction; (**e**) HOG features.

Before HOG features extraction for apple images, the appropriate apple images block size need to be selected. The setups of the block size in this work were referred to Zhao et al.^36^ providing a practical guidance for image identification. The blocks size was 4 × 4. If the block size was too large, the feature extraction was missing and the feature expression was blurred. If the block size was too small, the excess of useless interference information was collected with computational complexity increasing. With the parameters were selected, the horizontal gradient map and vertical gradient map of the apple images were calculated. After calculation, the images were divided into cell units and a histogram of gradient directions were constructed. A block was composed of 4 × 4 cells and the normalized gradient histogram was obtained within the block. The HOG feature of the image was obtained by concatenating the features of all blocks. Extraction of structural features using HOG were shown in Fig. 12.

GLCM and HOG features were merged as the input of SVM classifier^37^. Confusion matrix, a supervised classification learning algorithm, was selected to evaluate the accuracy of the classification results for apple quality. The accuracy can be described as Eq. (12):

12 $$ A = \frac{{N_{T} }}{{N_{V} }} \times 100\% , $$

where *A*, $N_{T}$ and $N_{V}$ represent accuracy of apple quality classification, the number of correct classification, and total number of validation datasets, respectively.

The training time for traditional method was 287 min. After training process, 498 and 23 images of premium apple samples were considered as middle apple class and poor apple class, respectively. 213 and 451 images of middle apple samples were considered as premium apple class and poor apple class, respectively. 21 and 368 images of poor apple samples were considered as premium apple class and middle apple class, respectively. The classification results of validation set using traditional method, which combined GLCM and HOG features and SVM classifier, were shown in Table 2. The SVM classifier was developed based on the GLCM and HOG features to distinguish apple quality with the overall accuracy of 78.14% for validation data set.

**Table 2:** Classification results of validation set for traditional method.

Actual classification	Predicted classification		
	Premium	Middle	Poor
Premium	1879	498	23
Middle	213	1736	451
Poor	21	368	2011

### Testing and discussions

**Figure 13:**
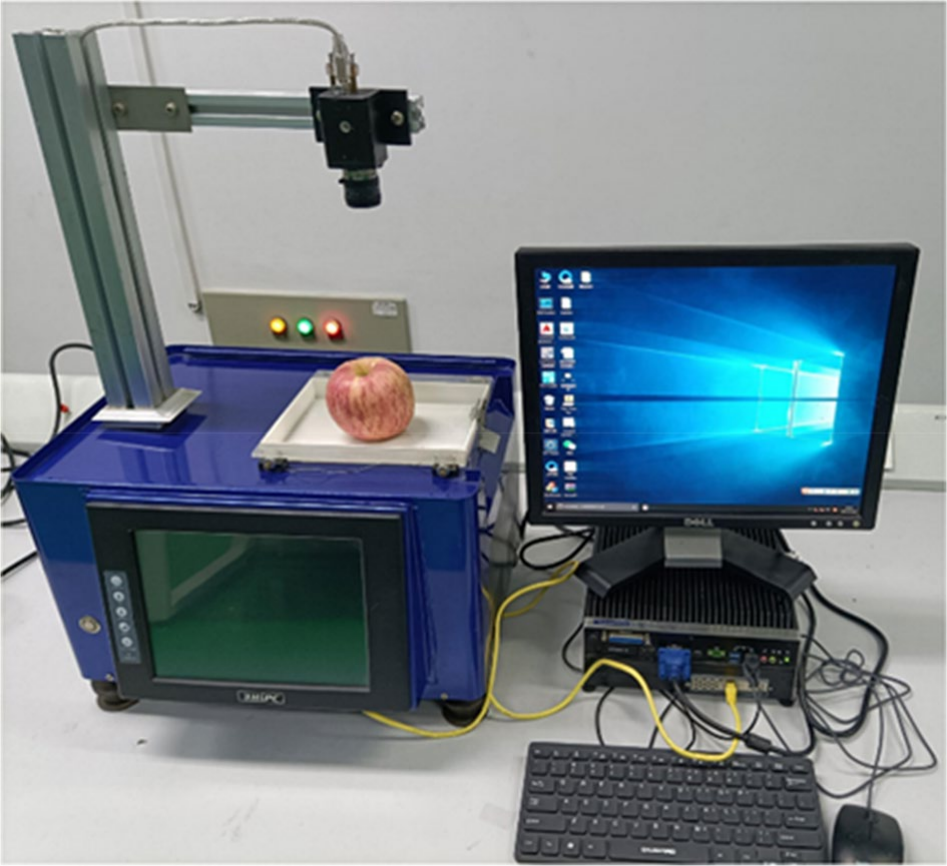
The online detection system for apple quality detection.

**Figure 14:**
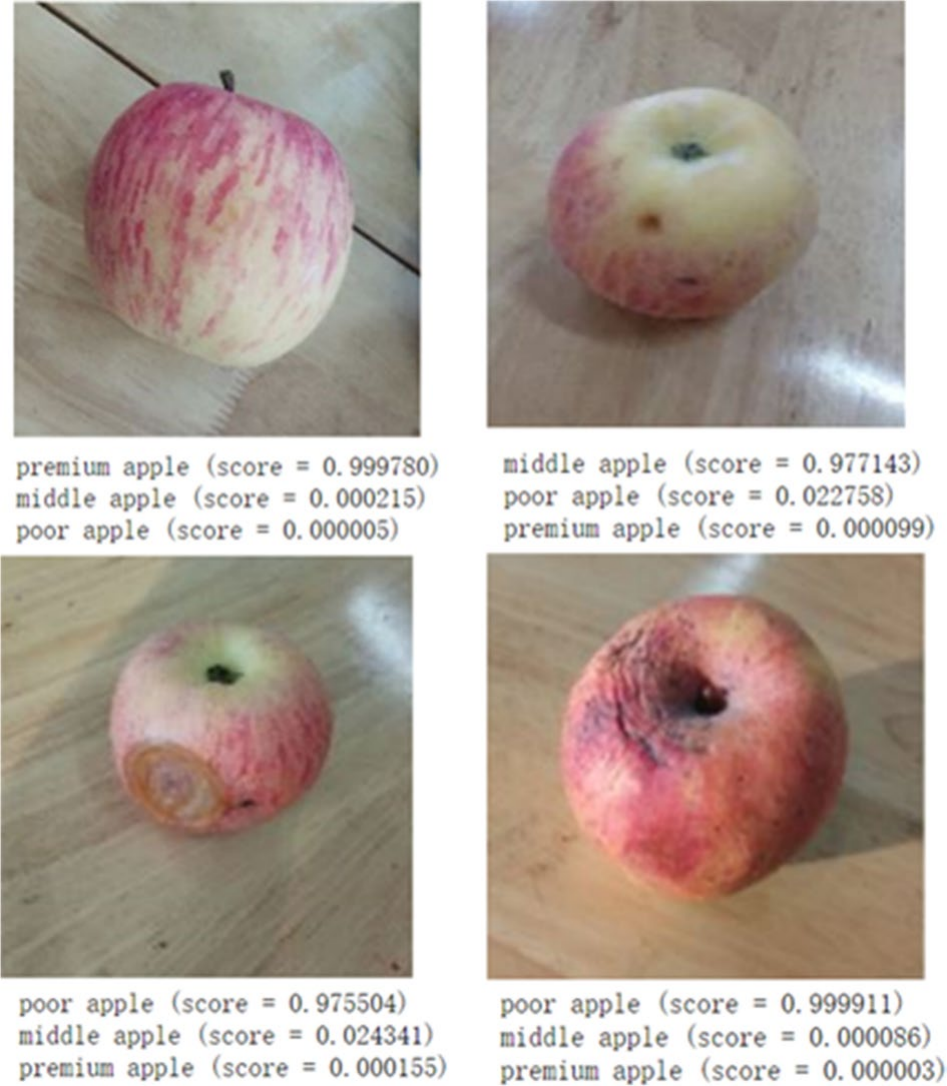
A few of prediction results for independent testing dataset.

**Figure 15:**
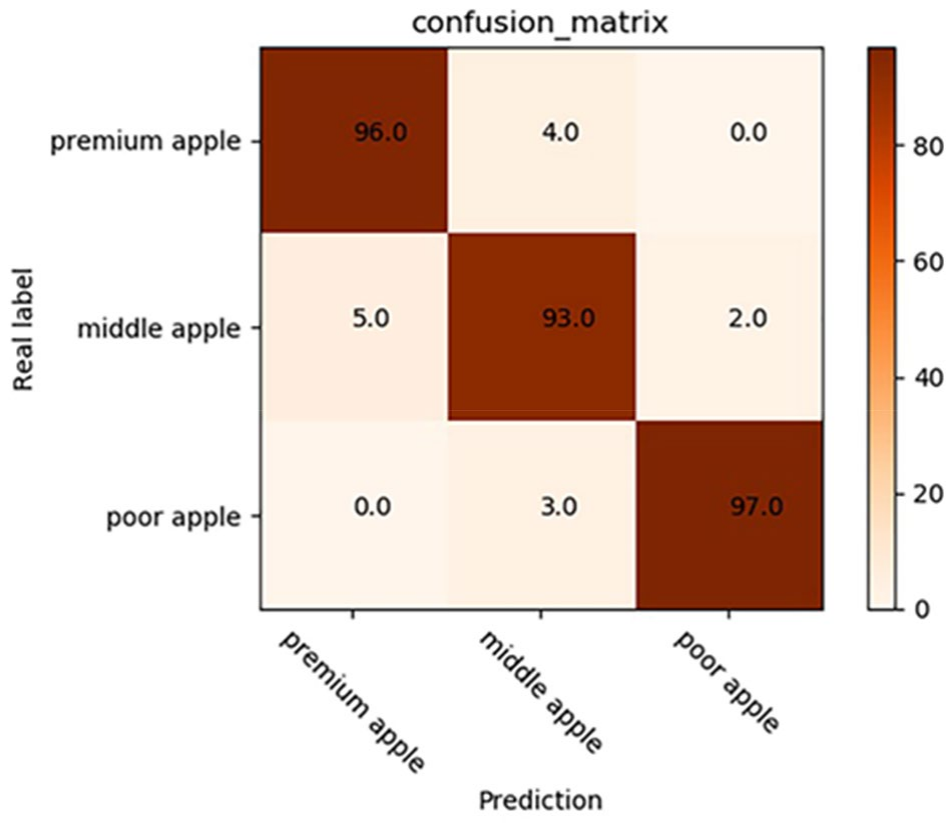
Confusion matrix of proposed model for the separate testing dataset.

Compared with Google inception v3 model and traditional image processing classification method, the proposed model obtained satisfying performance^38^. Therefore, a software was developed for image acquisition using Python and PyQt5 on Windows system. OpenCV and camera’s API were integrated into this software by Python language employing to acquire and save images. The weights, biases, and structure of the trained proposed model were saved and converted to a Protobuf format file. this file was loaded the software to realize online detection and classification for apple quality. The online detection system was shown in Fig. 13. Simultaneously, an independent testing dataset (300 apples with 100 for each class) was established to test the performance of the proposed model in online detection system. In the test experiment, four images of every apple were acquired by the camera at different angles. Then these images were predicted and scored by the trained proposed model. Some results of prediction and score were shown in Fig. 14. The quality category (premium apple, middle apple, and poor apple) with the highest total score was considered to be the predicted result. The proposed model demonstrated excellent performance for the separate testing dataset. The performance of proposed model was assessed the assistance of a confusion matrix in Fig. 15. In confusion matrix, 96 premium apples were rightly identified and 4 premium apples were considered as middle apples. 5, 93, and 2 middle apples were shown as premium, middle and poor apples, respectively. 97 poor apples were correctly classified and 3 poor apples were recognized as middle apples. The overall classification accuracy of proposed model for testing set was 95.33%. The trained Google Inception v3 model was also loaded into the software to test the performance with overall accuracy of 91.33% for separate testing dataset. Meanwhile, the trained SVM classifier was tested to distinguish apple quality with the overall accuracy of 77.67% for independent testing dataset.

Although the detection and classification accuracy will be affected by the complicated working environment such as Apple’s moving speed, the number, performance and angle of cameras^39^, the detection and classification results obtained by proposed model were superior to spectral imaging technology^9,11,12,40^ and traditional machine vision method^22,23^. Feature learning was an advantage of deep convolutional networks over traditional image processing method. Zhang et al.^41^ researched blueberry bruising using VGG-16 model (popular architectures). Due to the large number of layers and training parameters of the popular framework, the calculation time did not meet the requirements of Apple's detection and classification. Therefore, a new model for apple detection and classification was proposed in this article.

## Conclusions

In this paper, a novel method based on Convolutional Neural Networks (CNN) was proposed and employed for apple quality classification containing disturbing background. Three methods of proposed model based on CNN, Google Inception v3 model (popular architectures) and HOG/GLCM + SVM (traditional imaging process method) were trained and validated to identify apple quality. The proposed model was trained and validated with best training and validation accuracy of 99% and 98.98%, respectively. The greatest accuracy generated by Google Inceptionv3 model in training and validation were 92% and 91.2%, respectively. The SVM classifier was trained based on the GLCM and HOG features to distinguish apple quality with the overall accuracy of 78.14% for validation data set. The proposed model was more acceptable than the other two methods from the accurate results. In addition, the training time of proposed model, Google Inception v3 model and HOG/GLCM + SVM were 27, 51, and 287 min, respectively. The proposed model took the shortest times for training process. Moreover, three methods were tested using independent testing set, obtaining the accuracy of 95.33%, 91.33%, and 77.67%, respectively. The overall results showed that the proposed model has great potential in apple quality detection and classification. The proposed model detects more apple quality attributes including color, size, types, ripe or unripe, and physiological disorders in the future. The proposed model can be further extended to identify more than three categories of apple quality and classify other fruits. The proposed model will be deployed real online sorting equipment to test it performance in the future.

## Abbreviations

ANN: Artificial neural network
ASM: Angular second moment
CNN: Convolutional neural networks
CON: Contrast
COR: Correlation
DA: Discriminant analysis
ENT: Entropy
GLCM: Gray level co-occurrence matrix
HOG: Histogram of oriented gradient
HIS: Hyperspectral imaging
ILSVRC: ImageNet large scale visual recognition challenge
MNF: Minimum noise fraction
NIR: Near-infrared
PLS: Particle least square
SPA: Successive projections algorithm
SVM: Support vector machine
SWIR: Shortwave infrared
